# Shifting Paradigms in Hemorrhoid Management: The Emergence and Impact of Cap-Assisted Endoscopic Sclerotherapy

**DOI:** 10.3390/jcm13237284

**Published:** 2024-11-29

**Authors:** Xianglu Wang, Xia Wu, Quan Wen, Bota Cui, Faming Zhang

**Affiliations:** 1 Medical Center for Digestive Diseases, The Second Affiliated Hospital of Nanjing Medical University, Nanjing 210011, China; wangxianglu@stu.njmu.edu.cn (X.W.); xiawu@njmu.edu.cn (X.W.); wenquan@njmu.edu.cn (Q.W.); cuibota@njmu.edu.cn (B.C.); 2 Key Lab of Holistic Integrative Enterology, The Second Affiliated Hospital of Nanjing Medical University, Nanjing 210011, China

**Keywords:** hemorrhoids, hemorrhoidal disease, injection sclerotherapy, rubber band ligation, cap-assisted endoscopic sclerotherapy, anus positioning

## Abstract

Hemorrhoidal disease (HD) is a prevalent proctological condition that has puzzled people since ancient times, and the most common symptom is painless bleeding. Traditional treatments include conservative treatment, nonsurgical office-based treatments, and surgery. Sclerotherapy is one of the oldest forms of nonoperative intervention and is widely used to treat internal hemorrhoids with the development of endoscopy technology. However, sclerotherapy is always accompanied by complications such as bleeding, pain, abscess, etc., when the sclerosant is injected into the wrong site. Cap-assisted endoscopic sclerotherapy (CAES), a new minimally invasive technology, was first time coined in 2015 for the treatment of hemorrhoidal disease. The left-posterior–right-anterior (LPRA) anus positioning method under endoscopy provides reliable methodological support for advancing hemorrhoidal treatment via endoscopy. The current trend is that treatment for HD has shifted from being performed predominantly by the Department of Proctology Surgery to being managed mostly by the Department of Gastroenterology. This review reviewed the shifting paradigms of sclerotherapy for HD and discussed the emerging development of CAES.

## 1. Introduction

Hemorrhoidal disease (HD) significantly impacts people’s daily lives. An epidemiological survey indicates that HD affects approximately 4.4% of the population in the United States [1], while the prevalence rate in China is 51.56%, with the highest occurrence between the ages of 35 and 59 [2]. Furthermore, HD presents a substantial economic burden; in 2014, 1.4 million individuals within the employer-insured population in the United States sought treatment for HD, resulting in an estimated annual cost ranging from USD 770 million to USD 2.4 billion [3]. The symptoms of HD, which include painless bleeding, perianal pain, pruritus, fecal seepage, and protrusion, continue to plague individuals’ daily lives. It is estimated that 50% of the population older than 50 years has experienced these problems [4].

HD is classified as external, internal, or mixed based on the site of the condition, and this review specifically focuses on the treatment of internal hemorrhoids. Internal hemorrhoids are located above the dentate line, and their treatment includes conservative treatments, nonsurgical office-based treatments, and surgery, which is guided by the Goligher classification. Lifestyle changes, particularly in bowel habits, are necessary in conservative treatments. Medications such as fiber supplements, stimulant laxatives, and osmotic agents have demonstrated consistent beneficial effects for HD [5,6]. Natural compounds combined with flavonoids have been shown to effectively manage HD in several clinical trials [7,8], providing more treatment options. Patients with Grade I–III internal hemorrhoids can choose office-based treatments including rubber band ligation, infrared coagulation, and sclerotherapy. Surgical removal of hemorrhoids is recommended for patients with mixed or Grade IV internal hemorrhoids who have not responded to outpatient treatment [9] and includes Milligan–Morgan hemorrhoidectomy and closed hemorrhoidectomy, among others. However, current treatment trends are shifting towards more minimally invasive procedures and shorter hospital stays, even for advanced hemorrhoids. It is noteworthy that some gastroenterologists now consider the symptom of HD as a primary criterion for treatment selection rather than relying solely on the Goligher classification. For example, sclerotherapy is recognized as an effective treatment for bleeding internal hemorrhoids, while rubber band ligation is thought to be more effective for managing prolapse [10,11].

With advancements in technology, endoscopic approaches, including endoscopic rubber band ligation (ERBL) and endoscopic sclerotherapy, have become widely used in clinical practice due to their convenience and decreased pain [12,13]. However, these procedures are always accompanied with significant complications, such as bleeding, abscess, and urinary symptoms [14,15], which may hinder the development of endoscopic therapy. Cap-assisted endoscopic sclerotherapy (CAES), a novel endoscopic technique, was developed to minimize iatrogenic injury by enhancing the visibility of the surgical field [16]; however, it has been performed only in China to date. This review aims to examine the history of HD treatment, the development of sclerotherapy, and the potential application of CAES in other countries.

## 2. The Development of Sclerotherapy

### 2.1. The Development of HD Treatment

**Figure 1:**
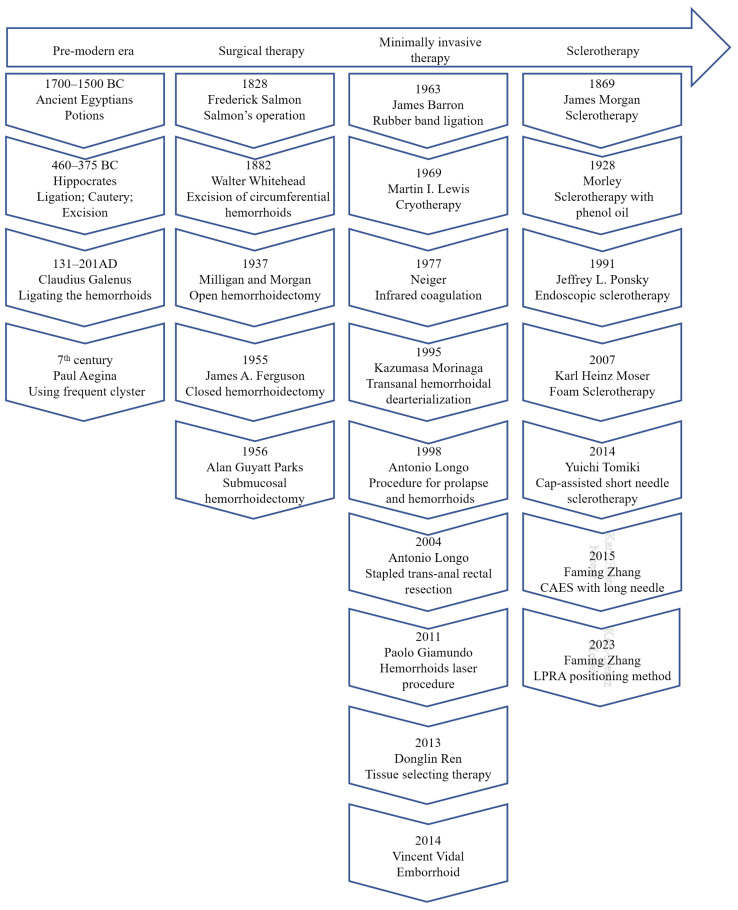
The history of hemorrhoid management [16,17,18,19,21,23,24,25,27,28,29,30,31,32].

The treatment of HD has been documented throughout recorded history (Figure 1). Ancient Egyptians described symptoms of painful swellings in the anus and treated them with potions containing honey, myrrh, flour, ibex fat, and sweet beer [17]. Hippocrates meticulously recorded the clinical manifestations and surgical interventions for HD in his treatise ‘On Hemorrhoids’, and the latter included ligation, cautery, and excision [18]. In Ancient Rome, Galen improved the existing surgical protocols. For the next thousand years, there were no significant advancements in the treatment of HD.

Since the 18th century, various surgical procedures have been developed to treat HD, including submucosal hemorrhoidectomy, anal stretching, Milligan–Morgan hemorrhoidectomy, and closed hemorrhoidectomy [19]. These procedures continue to be performed today. Surgical excision remains the gold standard for managing Grade III and IV internal hemorrhoids; however, postoperative pain remains a significant concern [20], although several pharmacological strategies for managing postoperative pain have been documented in the literature [4].

Office-based treatments have been introduced in clinical practice since the mid-19^th^ century. The injection of sclerosing agents was first utilized to treat HD in 1869 [19]. Blaisdell introduced rubber band ligation (RBL) in 1958, which was later refined by Barron in 1963 [21,22]. Cryotherapy and infrared coagulation were invented in the subsequent decades [23]; however, these methods were not adopted as widely as RBL and sclerotherapy. At the end of the 20^th^ century, two additional techniques were described: transanal hemorrhoidal dearterialization, performed by a designated proctoscope with a Doppler probe, and stapled hemorrhoidopexy, which aimed to preserve anal pad function [19,24]. Meanwhile, endoscopy was gradually applied for the treatment of internal hemorrhoids [25,26].

In the first two decades of the 21^st^ century, the hemorrhoid laser procedure and the emborrhoid technique were developed to minimize postoperative pain and reduce hospital stays [27,28]. Based on endoscopic sclerotherapy with cap assistance [29], our team introduced the CAES in 2015, which has gained widespread use in China [16].

### 2.2. The Development of Sclerosing Agents

In 1869, a surgeon named James Morgan first described injection therapy for HD using iron sulfate in Dublin. Subsequently, in 1871, Mitchell used carbolic acid as the injecting agent, which facilitated the advancement of sclerotherapy in the United States [30]. During a meeting of the Chicago Medical Society in 1879, Andrew Edmunds reported adverse events resulting from sclerotherapy involving the use of carbolic acid and olive oil for HD. Among the 3295 patients, 9 cases, 5 cases, 10 cases, and 8 cases suffered death, dangerous bleeding, abscess formation, and hepatic artery embolization, respectively. Furthermore, it was noted that at least 25% of the patients suffered severe pain [33].

In 1928, a radical change in sclerotherapy appeared: Morley injected 5% phenol oil into the submucosal space above the piles. Dukes subsequently reported that phenol oil can induce aseptic inflammation within the submucosa, leading to the formation of fibrous tissue that constricted blood vessels and resulted in the atrophy of the hemorrhoidal tissue [30]. Sclerotherapy with phenol oil can effectively minimize the risk of severe complications associated with necrosis of the hemorrhoidal core, which can occur with the application of carbolic acid and olive oil. As a result, 5% phenol oil became the most used agent in sclerotherapy, although other agents such as urethane, nitric acid, iodine, alum, and quinine were also used [34]. Sclerotherapy has been proven to be an effective and safe treatment for HD [35,36]. In China, a novel sclerosing agent named Xiaozhiling was introduced by Shi Zhaoqi in 1977 based on traditional Chinese medicine and has been regarded as a relatively ideal nonoperative approach for the treatment of HD [37]. Entering the 21st century, Shaobei injection has emerged as another widely used traditional Chinese medicine for the management of HD [38].

Sclerotherapy underwent a new stage of development with the application of sclerosing foam in 2007, when Moser first used 3% polidocanol foam as a sclerosing agent [31]. In a subsequent study, he found that approximately 88% of patients were treated successfully in the polidocanol foam group, compared to 69% in the oil-based agent group. This was also associated with increased patient satisfaction in the polidocanol foam cohort. With the proven safety and efficacy [39,40,41], polidocanol foam has been increasingly adopted worldwide. In Japan, a novel sclerosant called aluminum potassium sulfate and tannic acid (LATA), simplified from Xiaozhiling, showed better efficacy than 5% phenol oil [42]. Multiple studies have confirmed that injection sclerotherapy using LATA is effective even for Grade III–IV hemorrhoids [43,44,45]. Notably, the cumulative success rate in patients with Grade III hemorrhoids reached 96.5% after one year of ALTA therapy combined with rectal mucopexy [44]. In China, since 2014, Lauromacrogol has been extensively used for the treatment of Grade I–III internal hemorrhoids [16,46].

### 2.3. The Development of Endoscopic Sclerotherapy

In addition to the changes in sclerosing agents, injection sclerotherapy has significantly improved the management of hemorrhoidal disease in recent decades. In 1991, Ponsky first attempted to treat internal hemorrhoids by employing retrograde endoscopy to inject 23.4% hypertonic saline [25]. Benin subsequently reported on the application of endoscopic injection in 250 patients with Grade II–IV internal hemorrhoids treated with sodium tetradecyl sulfate. The findings indicated that bleeding, pain, and hemorrhoidal prolapse were resolved in all patients within a maximum of two sclerotherapy sessions, with no reported complications such as erosions, abscesses, bacteremia, or fistulas [47]. The postprocedural adverse events associated with proctoscopy are comparable to those observed with colonoscopy in injection sclerotherapy, as are the therapeutic effects and patient satisfaction [48]. Previous procedures were performed with a rigid anoscope, which presented a blind area that hindered precise operation. Misplaced injections could result in iatrogenic risks, including pain, perianal abscess, impotence, and other complications [49]. The development of flexible reversible endoscopes has enhanced imaging capabilities and increased controllability, allowing for greater interventional precision, and reducing iatrogenic risk. Additionally, colonoscopy can identify and enable simultaneous treatment of potential HD during routine colon cancer screening [16,32]. Table 1 provides a summary of significant studies related to injection sclerotherapy.

**Figure 2:**
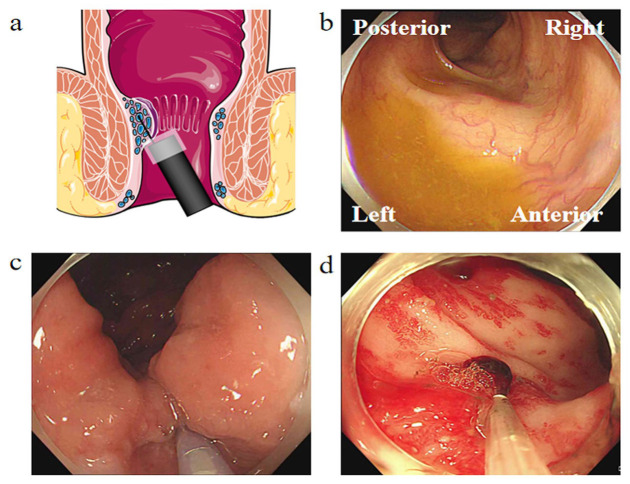
Cap-assisted endoscopic sclerotherapy. (**a**) Diagrammatic drawing of CAES for internal hemorrhoids. (**b**) LPRA anus positioning methods under flexible endoscopy. (**c**) Submucosal injection sclerotherapy for internal hemorrhoids. (**d**) Small-dose injection of sclerotherapy for proctorrhagia caused by radiation enteritis.

In 2014, Tomiki reported the flexible endoscopic injection of ALTA with a short needle and cap assistance in Japan [29]. Considering the limitations of retrograde endoscopy during injection sclerotherapy within the anus, we first conducted the cap-assisted endoscopic sclerotherapy with a long injection needle for patients with Grades I–III hemorrhoids in 2015 (Figure 1 and Figure 2a) [16]. In 2021, an expert panel in China developed guidance on CAES and introduced the Left-Posterior—Right-Anterior (LPRA) positioning method for anal procedures [32]. The presence of residual fluid or injected water within the anal cavity, as observed during endoscopy, serves as an indicator for identifying the left side of the anus when the patient is positioned in the left lateral decubitus position. Along the clockwise direction, the LPRA positioning method is recommended as a replacement for the conventional lithotomy position for the precise direction description on the anal lesions and endoscopic therapy (Figure 2b). This LPRA positioning technique is suitable for application in endoscopic procedures and has the potential to enhance clinical workflows and scientific communication. Both CAES and the LPRA positioning method has garnered significant attention on a global scale.

## 3. The Development of CAES

### 3.1. The Procedure of CAES

The methodology of CAES was initially documented in 2015. A short, straight, transparent cap is installed at the front end of the endoscope, and an appropriate amount of gas is insufflated into the rectum to maximize visibility of the targeting field [16]. Injection sites were selected for an endoscopic direction of 6 o’clock, and the clockwise order should be followed. The LPRA positioning method could help endoscopists locate injected and non-injected sites. While the use of a color tracer is generally discouraged, it may be beneficial for less experienced endoscopists [32]. The sclerosing agent should be injected into the submucosal layer within 5 s, and very quick injection or more than 2 mL injection at one site is not permitted. After injection sclerotherapy, the needle should be kept stable without moving for at least 5 s to prevent bleeding. After retracting the endoscope, a finger massage around the anal ring should be performed to help disperse the sclerosing agents. Neither antibiotics nor hemostatic agents are required during the operation period. In China, the sclerosing agents mainly include lauromacrogol and traditional Chinese medicine (e.g., Shaobei or Xiaozhiling), whereas ALTA is predominantly used in Japan. Although a long needle is recommended for HD accompanied by prolapse, both long and short needles can be selected for bleeding hemorrhoids [32]. Finally, health education is crucial in preventing the recurrence of HD.

### 3.2. The Clinical Application of CAES

CAES is an innovative flexible endoscopy technique that is superior to traditional injection sclerotherapy in the following aspects. First, a short and straight cap used in endoscopic submucosal dissection is attached to the top of the colonoscope, and enough air is injected into the rectum. These operations can maximize the visibility of the endoscopic view and avoid reverse examination and treatment. Second, a long needle (length ≥ 10 mm) is used for the injection of the sclerosing agent instead of a short needle [32], which could accurately control over the injection angle, direction, and depth under direct vision to avoid iatrogenic injury. Finally, endoscopists can perform simultaneous endoscopic diagnosis and therapy based on the same colon preparation procedure and save the related medical costs and alleviate physical and mental pain for patients. Additionally, CASE is similar to existing endoscopic techniques, and gastroenterologists can easily master the relevant technologies [55].

The length of the needle used in injection sclerotherapy remains controversial. The short needle (length 3–5 mm) was suggested for traditional sclerotherapy to avoid its insertion into dangerous areas [52]. However, using a short needle in CAES may lead to additional mucosal injury, potential inflammation, and complications such as artificial ulcers and secondary bleeding during multiple-site injections due to its short length. A long needle may overcome the above shortcomings. Nonetheless, there is currently no solid clinical evidence for this. Consequently, we have designed a multicenter randomized controlled trial to evaluate the efficacy and safety of CAES with long or short injection needles (NCT03917056).

The indications for CAES for HD include patients with symptomatic Grade I–II internal hemorrhoids when conservative management is ineffective, as well as patients with Grade III internal hemorrhoids who are unsuitable for surgery or refuse surgical intervention. CAES is also an option for treating symptomatic rectal mucosal prolapse (Figure 2c) [56]. Furthermore, CAES may be beneficial for patients with active proctitis, radiation enteritis, immune-related ulcers, or unexplained anal ulcers; however, conventional-dose injections are not recommended [32]. Additionally, during CAES, endoscopists can perform a colonoscopy to distinguish better bleeding from other anorectal symptoms, and possible polypectomy, excision of anal papilla fibromas, and biopsy of the polyps could also be carried out.

For patients with immunodeficiency, cerebrovascular accidents, or hypercoagulability disorders, CAES may be an option for bleeding hemorrhoids during an emergency after balancing possible benefits and potential risk from the interventional procedure. However, CAES is not recommended to patients with perianal abscesses, strictures, fistulas, anal malignancies, and pregnancy. CAES procedure-associated complications mainly include difficulties in passing gas, bleeding, infection, ulcer, and chronic anal pain, which were mainly reported by physicians who were in the early stages of CAES training [57]. Other rare complications were reported in some clinical studies, such as hematuria, urinary retention, urethral stricture, impotence, and septicemia [58,59].

In the initial clinical trial, all patients achieved the expected clinical response and were satisfied with CAES, with no obvious complications. Notably, only one patient presented with mild tenesmus within four days after CAES, which was associated with one injection site below the dentate line [16]. In 2022, Xie attempted to assess the long-term efficacy and safety of CAES with long injection needles for Grade I–IV internal hemorrhoids [46], and reported that 62.7% of patients had both improved hemorrhoid grades and symptoms at the first median follow-up of 33 months post-CAES. At the second follow-up one year later, 61.7% of the patients reported satisfactory improvement. No complications occurred after CAES, including anal bleeding, anal fistula, or anal stenosis. These results suggested that CAES is a convenient, safe, and effective flexible endoscopic therapy for internal hemorrhoids. However, Zheng reported a case of thrombosis in a patient with Grade III hemorrhoids who was treated with CAES, which was presumably due to an inaccurate injection depth [60].

### 3.3. The Clinical Advantage of Endoscopic Injection Sclerotherapy and CAES

Current endoscopic approaches for HD mainly include ERBL and endoscopic injection sclerotherapy (EIS). Several systematic reviews and meta-analysis comparing RBL with sclerotherapy have indicated that RBL has an advantage in controlling prolapse and bleeding and increasing patient satisfaction; however, it is associated with significantly greater postprocedural pain [61,62]. A prospective study for Grade I–III internal hemorrhoid showed that both ERBL and EIS were equally effective in alleviating the severity with no significant differences in complication rates; nonetheless, the pain associated with ERBL was significantly greater than EIS [53]. Conversely, in a recent randomized controlled trial comparing RBL to injection sclerotherapy for Grades I–III internal hemorrhoids, the complete success rate was significantly higher in the polidocanol sclerotherapy group compared to the RBL group, with fewer repeat sessions and complications [54]. Additionally, another meta-analysis showed that patients undergoing sclerotherapy exhibited a higher therapeutic success rate compared to the RBL group [63]. Other office-based treatments, such as cryotherapy and coagulation, are also applied in clinical practice but are less prevalent than ERBL and EIS. Varma reported that injection sclerotherapy had higher early cure rates for bleeding and prolapse than ultrasonic coagulation for the treatment of HD with short operating time and greater convenience [64]. Another randomized controlled trial revealed that electrocoagulation was more effective than injection sclerotherapy in reducing rectal bleeding, but it caused more pain for patients [65]. However, these results should be a reference for internal hemorrhoid treatment because these studies are heterogenous in hemorrhoidal grades, sclerosants, injection volumes, and others. Overall, both ERBL and EIS are safe and feasible first-line treatments for HD, and the combination of ERBL and EIS could increase the curative effect and reduce the risk of delayed bleeding and abscess [50,66]. RBL, either endoscopy or anoscopy, has largely replaced sclerotherapy for treatment of small internal hemorrhoids in Western countries. However, gastroenterologists have fundamentally changed the trend of hemorrhoid treatment through CAES under endoscopy in recent years in China, due to the advantages of less pain, expedited recovery, and shorter procedure time [16,46]; this operation may be the future development trend of global hemorrhoid treatment.

Patients typically decline hemorrhoidectomy because of the long hospital stays, postoperative pain, and potential complications, even if hemorrhoidectomy has a higher success rate than sclerotherapy [67,68]. Sclerotherapy causes less pain and fewer serious complications, making it a viable alternative to surgical intervention [43]. Moreover, the duration of the operation is significantly shorter for sclerotherapy than for hemorrhoidectomy; a meta-analysis reported that the median procedural time for sclerotherapy was merely 8.5 min across 11 clinical studies [69]. During the COVID-19 epidemic in Italy, patients with Grade III–IV hemorrhoids who could not receive surgery were treated with 3% polidocanol foam sclerotherapy. All patients were discharged 10 min after the treatment without experiencing any adverse events and resumed their normal daily activities the following day [12]. Furthermore, the combination of injection sclerotherapy and hemorrhoidectomy could also reduce overall recurrence rates compared to injection sclerotherapy alone [70,71]. The etiology of HD is multifactorial, including sliding anal cushions, vascular abnormality, rectal redundancy, and elevated resting anal pressure. Stapled hemorrhoidopexy, or procedures for prolapse and hemorrhoids, are resolutive treatments of HD based on the theory of rectal redundancy; however, it is noteworthy that injection sclerotherapy is supported by the multifactorial nature of hemorrhoidal disease [72]. In general, EIS represents an excellent therapeutic option for the management of internal hemorrhoids and rectal mucosal prolapse.

### 3.4. The CAES in Special Populations

Symptomatic hemorrhoids may occur in patients with inflammatory bowel disease (IBD) as a consequence of chronic diarrhea [73]. Conservative treatments are recommended for patients with active inflammation and perianal skin tags. A meta-analysis involving 135 patients treated with hemorrhoidectomy revealed that the risk of complications was higher in patients diagnosed with Crohn’s disease compared to those with ulcerative colitis. Furthermore, complications were observed less frequently in patients with a known diagnosis of IBD [74]. For patients whose IBD is in remission and for whom conservative managements are not effective, transanal hemorrhoidal dearterialization, hemorrhoidectomy, and RBL are options.

Conservative treatments are typically the preferred choice for patients with HD combined with coagulation dysfunction. However, RBL is not recommended for patients taking anticoagulants because warfarin and clopidogrel could increase the risk of post-procedural bleeding following RBL [75,76]. A case-matched study proved that antithrombotic treatment does not significantly increase the incidence of postoperative complications during ALTA injection sclerotherapy, with a similar efficacy in patients with bleeding [77]. Consequently, injection sclerotherapy is strongly recommended for patients with difficulty stopping antithrombotic therapy. Additionally, decompensated cirrhosis increases the risk of surgical therapy in cases with hemorrhoidal bleeding; however, sclerotherapy is an ideal alternative, even for high-risk patients [51,78].

Injection sclerotherapy may provide a safer alternative for symptomatic hemorrhoids in immunodeficient patients. Previous studies have indicated that the incidence of wound and infectious complications after hemorrhoidectomy or RBL is increased in patients with acquired immunodeficiency syndrome (AIDS) [79]. Scaglia reported that no complications occurred in 22 AIDS patients treated with injection sclerotherapy for bleeding induced by Grade II–IV hemorrhoids [80]. Therefore, injection sclerotherapy may be considered the preferred treatment option for patients with AIDS due to the high risk of surgery, and additional clinical studies are necessary to support it.

The incidence of HD is extremely rare in children, with a prevalence of one to two cases annually [81]. A retrospective study of 14 pediatric patients reported that sclerotherapy with polidocanol is a safe and effective treatment for children, resulting in less invasive injury [82]. Furthermore, injection sclerotherapy could be an adjunctive treatment to distal hemorrhoidectomy for mixed hemorrhoids with a 100% remission rate for prolapse symptoms [71].

### 3.5. The Future Trend of CAES

Since the introduction of the CAES in 2015, the disciplinary landscape for HD has altered in China. The era of endoscopic, minimally invasive technologies is changing the treatment paradigms for many diseases, from surgery to internal medicine, with HD being a notable example [83]. Compared to traditional treatment methods, CAES has the advantages of less pain, expedited recovery, and shorter procedure time. Consequently, the management of HD in China has shifted from being predominantly managed by the department of proctology surgery to being managed mostly by the department of gastroenterology. With the expansion of research, CAES would be a preferred treatment method for HD globally [84].

Although CAES was originally invented to avoid complications of HD, due to the advantage of expanding maximal visibility, it can be applied to other gastrointestinal disorders in the future. In addition to internal hemorrhoidal bleeding, CAES presents a potential therapeutic option for gastrointestinal hemorrhage. Excessive colorectal hemorrhage caused by tumors, vascular malformations, radiation enteritis, and other conditions can be life-threatening. Quick hemostasis is critical, especially when the underlying condition does not show rapid improvement. Moreover, CAES could protect the patient’s life by effectively halting bleeding. CAES for colorectal hemorrhage caused by radiation enteritis is shown in Figure 2d.

CAES has been demonstrated to be an effective treatment for patients with esophageal varices. A retrospective study involving 95 patients with cirrhosis and esophageal variceal bleeding reported that the volume of sclerosant, mean number of sessions, endoscopic treatment time, and the time required for the initial eradication of esophageal varices were significantly lower in the CAES group compared to the direct EIS group, with a higher probability of variceal recurrence and rebleeding in the direct EIS group [85]. In another study that compared CAES with endoscopic variceal ligation for esophageal varices, the authors described that CAES can reduce the recurrence rate with comparable safety in long-term management [86].

## 4. Conclusions

The treatment of HD has evolved over hundreds of years with the progressed understanding of anatomy and etiology, invasive surgeries, sclerotherapy agents, and endoscopic procedures. In the endoscopy era, due to its lower pain and convenience, CAES is the preferred treatment for Grade I–III internal hemorrhoids, rectal mucosal prolapse, and other gastrointestinal disorders. Moreover, CAES has been reported to be effective in treating gastrointestinal hemorrhage and esophageal varices. However, we should acknowledge that this review is a literature review rather than a systematic review, due to the limited studies of CAES. More studies should be carried out to validate the efficacy and safety of CAES and promote the clinical application of CAES.

## Figures and Tables

**Table 1:** Studies of injection sclerotherapy.

Researcher	Agents	Approach	Number	Classification	Outcome	Complication
Andrew Edmunds [33]	Carbolic acid or olive oil	Anoscope	3295	N/A	N/A	9 cases of death, 5 cases of dangerous bleeding, 10 cases of abscess formation, and 8 cases of embolism to the liver, and more than about 25% suffered severe pain.
Ponsky et al. [25]	23.4% hypertonic saline	Colonoscope	19	I–III	17 patients had benign immediate post-procedure courses without serious complications.	One patient had acute rectal mucosal, and 2 patients complained of significant rectal pain.
Kanellos et al. [50]	Phenol oil	Colonoscope	85	II	6 patients were symptom free.	24 patients had minor complications and only one patient suffered severe pain.
Tokunaga et al. [43]	ALTA	Anoscope	784	II–III	Disappearance rates of prolapse were 96% in sclerotherapy.	Significant postoperative pain (needed injection of pain killer) occurred in 1.8% patients in sclerotherapy.
Awad et al. [51]	ethanolamine oleate 5% or N-butyl cyanoacrylate	Colonoscope	60	II–IV	EIS was highly effective in controlling bleeding with a low rebleeding (13.3%).	37 patients needed pain management.
Moser et al. [31]	Polidocanol foam or liquid	Proctoscope	130	I	88% of patients were treated successfully in polidocanol foam set compared to 69% in polidocanol liquid set after one sclerotherapy session.	24% of patients suffered pain in polidocanol foam set compared with 36% in polidocanol liquid set after one sclerotherapy session, and 1 case with acute prostatitis in foam group.
An et al. [38]	Shaobei or Xiaozhiling	N/A	1520	I–III	The efficacy rate was 97.5% in the Shaobei group, but 91.8% in the Xiaozhiling group.	The recurrence rate in the Shaobei group was 0.5%, with 1.3% in the Xiaozhiling group.
Tomiki et al. [52]	ALTA	Colonoscope	83	II–IV	Cure, improvement, and failure were observed in 54, 27, and 2 patients.	Fever was observed in 3 patients, with hematuria in 1 patient.
Yano et al. [42]	ALTA or phenol oil	Anoscope	135	III	The efficacies of ALTA and phenol oil one year after treatment were 75% and 20%.	N/A
Zhang et al. [16]	Lauromacrogol	Colonoscope (CAES)	30	I–III	All patients were satisfied with CAES.	One patient felt mild tenesmus within four days.
Tomiki et al. [48]	ALTA	Proctoscope or Colonoscope	81	II–III	The cure, improvement, and failure rates were 30.3%, 66.7%, and 3% in proctoscope group compared to 18.7%, 77.1%, and 4.2% in colonoscope group.	Post-procedural adverse events were observed in 4 patients (proctoscope) and 6 patients (colonoscope), including mild fever, anal pain, urination disorders, and ulcers.
Makanjuola et al. [53]	Polidocanol	Proctoscope	37	I–III	EIS was effective and safe in patients with internal hemorrhoids.	Ulceration was observed in 3 patients.
Mishra et al. [39]	Polidocanol or phenol oil	N/A	150	I–II	The success rates after the first session with polidocanol and phenol oil was 60.6% and 38.1% and 94.7% and 84% after the second session.	In the polidocanol group, 11.3% of the patients suffered pain compared to 17.5% in the phenol group.
Lisi et al. [12]	Polidocanol foam	N/A	10	III–IV	Bleeding and itching were relieved in all patients.	No complications occurred.
Lobascio et al. [40]	Polidocanol foam	Anoscope	66	II–III	The overall success rate was 78.8% after a single sclerotherapy session and 86% after two sessions.	No intraoperative complications and no drug-related side effects occurred.
Salgueiro et al. [54]	Polidocanol foam	N/A	60	I–III	The therapeutic success rate and complete success rate was 93.3% and 88.3%.	No severe complications were observed.
Abe et al. [45]	ALTA	Anoscope	1180	II–IV	Recurrence rates at 3, 6, and 9 years were 7.4%, 27.2%, and 47.5%.	Fever in 16 patients, rectal ulcer in 10 patients, rectal stricture in 5 patients, and perianal abscess in 4 patients.
Xie et al. [46]	Lauromacrogol	Colonoscope (CAES)	201	I–IV	62.7% of patients had satisfactory improvement in hemorrhoid grade and symptoms at the first follow-up 3 months postoperation, with 61.7% at the second follow-up.	No complications occurred after CAES with a long needle.
Gallo et al. [41]	Polidocanol foam	Anoscope	183	II	The overall success rate was 95.6% at 1 year, with 90.2% after the final 3-year follow-up.	3 episodes of external thrombosis, and no serious adverse events occurred.
Tsunoda et al. [44]	ALTA	Proctoscope	161	III	Therapeutic success rates at 1, 3, and 5 years were 96.5%, 85.3%, and 85.3% in patients treated by ALTA therapy with rectal mucopexy.	4 patients of intraoperative transitory submucosal, 1 patient of urinary retention, and 1 patient of thrombosed residual hemorrhoids.

Abbreviations: ALTA, aluminum potassium sulfate and tannic acid; N/A, not applicable; CAES, cap-assisted endoscopic sclerotherapy.

## Data Availability

No new data were created or analyzed in this study.
